# Healthcare utilisation among people living with sickle cell disease in the Upper West Region of Ghana

**DOI:** 10.1186/s12913-025-13124-7

**Published:** 2025-08-25

**Authors:** Clement Luciano Momore, Farrukh Ishaque Saah, Hubert Amu, Andrews Adjei Druye

**Affiliations:** 1 Department of Nursing, School of Nursing and Midwifery, College of Health and Allied Sciences, University of Cape Coast, Cape Coast, Ghana; 2 Department of Population and Health, Faculty of Social Science, College of Humanities and Legal Studies, University of Cape Coast, Cape Coast, Ghana; 3 Department of Population and Behavioural Science, FN Binka School of Public Health, University of Health and Allied Sciences, Hohoe, Ghana

**Keywords:** Sickle cell disease, Healthcare, Accessibility, Utilisation, Upper West Region, Ghana

## Abstract

**Background:**

Sickle Cell Disease (SCD) accounts for significant mortalities and hospitalisations in Ghana. It is characterised by acute and chronic complications that often require immediate and easy access to appropriate healthcare services, including emergency, in-patient, and non-preventive care. Poor access to healthcare services and healthcare-seeking behaviour are responsible for most SCD-preventable deaths. The determinants of healthcare service use among people living with SCD should be identified and addressed. Thus, the study investigated the accessibility and utilisation of healthcare services and its determinants among people living with SCD in the Upper West Region.

**Methods:**

This cross-sectional study involved 248 people with SCD in the Upper West Region accessing healthcare at the Wa Municipal Hospital’s Sickle Cell Unit, selected using a systematic random sampling technique. Data collected were collected using a pretested questionnaire and analysed using SPSS v22.0. The analysis included descriptive statistics, a chi-square test, and a logistic regression test at a 95% confidence interval with significance set at *p* < 0.05.

**Results:**

58% had poor access to healthcare facilities providing SCD care, and 92% had used healthcare services regularly within the last 12 months. Health problems responsible for most hospital visits were painful crises (96.5%), fever (96.0%), acute respiratory problems (85.1%), pneumonia and influenza (74.2%), and acute otitis media (32.6%) in the last 12 months. Service utilisation was predicted by being aged 40 + (AOR = 12.6, 95%CI = 1.40–113.81) and considering the distance to health facilities as very close (AOR = 0.03, 95%CI = 0.00–0.98).

**Conclusions:**

There was significantly poor access to healthcare facilities providing SCD care Healthcare service use was high and influenced by personal health problems and social factors. Healthcare infrastructure and training of specialists should be increased to improve accessibility, service availability, utilisation, and quality of care.

**Supplementary Information:**

The online version contains supplementary material available at 10.1186/s12913-025-13124-7.

## Introduction

Sickle cell disease (SCD) is the most common human genetic condition globally and affects 7.74 million people worldwide [1]. Between 2000 and 2021, it is estimated that global births of babies with SCD and people living with SCD increased by 13.7% and 41.4% respectively [1]. This highlights the increasing burden of SCD globally. In addition to the negative consequences of their medications, people with SCD endure the psychological and physical toll of recurrent periods of intense pain [2, 3]. Access to primary and specialised healthcare services must be continuous for both adults and children with SCD [2, 4]. While attempts to improve treatment have mostly concentrated on paediatric services, increasing children’s survival rates, adults’ access to and management of healthcare has received less attention [2]. Therefore, emergency care is often necessary for individuals with SCD, primarily for pain management [2, 5].

Sub-Saharan Africa (SSA) disproportionately accounts for these increases [1]. In most parts of the region, including Ghana, the burden of SCD is worsened by inadequate health infrastructure, poor nutrition and co‐morbidities of infections such as malaria, tuberculosis, and HIV, contributing to SCD-related mortalities [6, 7]. In higher-income countries, more than 90% of infants with SCD survive into adulthood due to available infrastructure for universal newborn screening and comprehensive care [6, 8]. Comprehensive care for SCD involves a multidisciplinary approach focusing on early diagnosis, patient and family education, and regular follow-up such as hydroxyurea, prophylaxis for bacterial infections and malaria, and appropriate use of blood transfusion, among others. It aims to prevent complications, manage crises, and improve the quality of life for individuals with SCD [9, 10]. These morbidity- and mortality‐reducing interventions associated with improved life expectancy are largely inaccessible to people with SCD in low‐income countries such as Ghana. SCD individuals in low- and middle-income countries therefore experience more frequent life-threatening complications like acute organ failure [6–8]. In these regions, almost 80% of babies born with SCD are undiagnosed [8]. Even among those diagnosed, misinformation and scepticism toward test results often hinder appropriate care uptake, further exacerbating healthcare accessibility challenges. As such, more than half (50% to 90%) die before age five, largely attributed to limited health service availability, access, and use [8, 11, 12].

SCD is characterised by recurrent complications that drive significant healthcare utilisation. One of the most severe manifestations is vaso-occlusive crises (VOC), where sickled red blood cells obstruct blood flow, leading to excruciating pain episodes, tissue damage, and potential organ dysfunction [2, 13]. VOCs often necessitate emergency care, hospitalization, and ongoing management, making individuals with SCD frequent users of healthcare services [2, 14]. Additionally, complications such as acute chest syndrome, infections, and anaemia further contribute to the substantial medical needs of people with SCD [15]. These complications compound over time contributing to high healthcare needs [16].

Optimal care is often achieved in clinics specialising in SCD. With easily accessible and appropriate healthcare services for SCD complications, these deaths may be prevented or greatly reduced [2, 3, 17]. The utilisation of healthcare services such as emergency care, in-patient for pain crises and severe complications, and outpatient services for routine check-ups are key in ameliorating common complications associated with SCD [3]. Without proper management, SCD can lead to severe complications, reduced life expectancy, and a diminished quality of life due to chronic pain, frequent hospitalizations, and organ damage [2, 14]. However, the determinants of healthcare utilisation among people living with the disease have been under-researched. Most studies have focused on the prevalence, epidemiology and general public knowledge of SCD [18–24]. Nevertheless, similar studies in Ghana have focused on: people with SCD’s knowledge about the disease; self-management strategies and recommendations for people with SCD; and general healthcare services utilisation [20, 24–29], most of which have employed mixed and qualitative methods and are from urban settings.

Wa Municipal Hospital, the only secondary-level healthcare facility in the Upper West Region, serves as the primary centre for SCD care. It provides regular outpatient SCD clinics for both paediatric and adult patients, with services including routine monitoring, pain management, hydroxyurea therapy, and health education. While hydroxyurea is available under Ghana’s National Health Insurance Scheme (NHIS) in selected tertiary facilities, it is not consistently accessible at district-level hospitals like Wa Municipal Hospital, potentially limiting treatment access for local patients. These clinics are conducted monthly for paediatric patients and every three months for adults, alongside emergency and inpatient care for severe complications. The facility records indicate that complications in people living with SCD frequently arise due to delayed care-seeking behaviour, citing 26 cases of late presentation, 20 instances of non-attendance at scheduled visits, and 16 cases of treatment defaulting. While non-attendance might reflect care-seeking elsewhere, Wa Municipal Hospital remains the primary point of SCD care in the region, seeing approximately 232 patients annually. No study has explored the determinants of healthcare utilisation among this group of people within the region.

There is therefore a knowledge gap in the literature that this study seeks to bridge by assessing the determinants of healthcare service utilisation among people with SCD, specifically, in the Upper West Region of Ghana. Healthcare utilisation among people with SCD and its determinants must be assessed due to the importance of access to and use of quality and appropriate healthcare to overall health status and quality of life. The study thus investigated the healthcare accessibility and utilisation of people living with SCD in the Upper West Region of Ghana.

### Conceptual framework

**Fig. 1:**
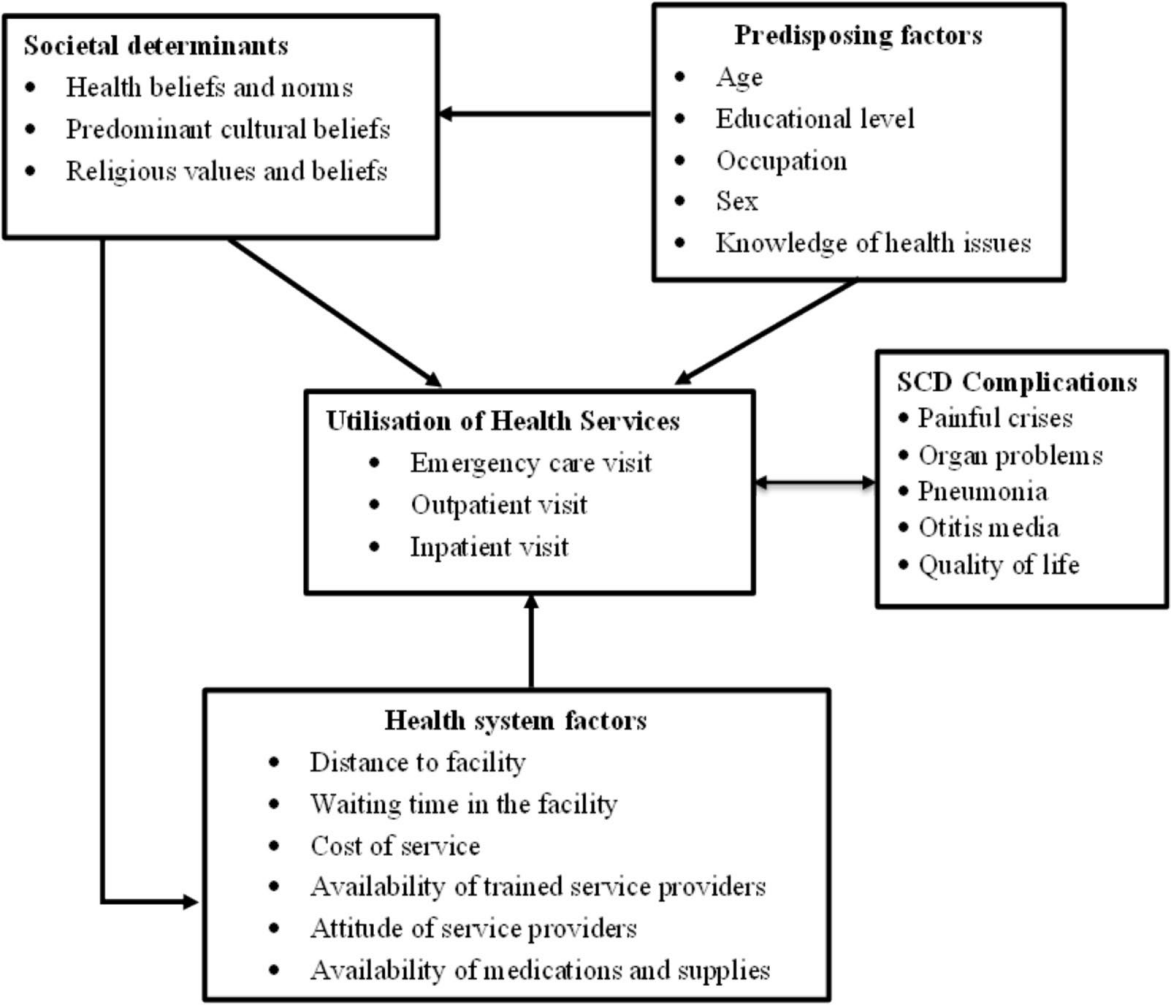
Conceptual framework on healthcare service utilisation. Source: Adapted from Andersen and Newman [32]

We adapted the Healthcare Utilization Model (HUM) as the conceptual framework (Fig. 1) to help explain variations in utilisation rates and uptake of health services. The HUM propounded by Andersen [30] is more appropriate in addressing all the components of healthcare utilisation and is a widely used model in literature for healthcare utilisation, hence, its adaptation as the model to underpin this study. Although some researchers have addressed the issue of healthcare usage, these studies did not adequately take into account influencing factors, contextual factors, or disease conditions [31]. The model thus provides a useful framework for examining how people utilise health services and organising the variables that have been linked to changes in that use. The model’s adaptation suggests prominent possible factors that may influence access and utilisation of healthcare services for managing complications and conditions associated with SCD. This model includes factors that influence the utilisation of health services, such as the attitude of healthcare providers, availability of specialist facilities and professionals, distance to a healthcare facility, sociocultural norms and beliefs, and cost involved in accessing and using healthcare services, among others, showcasing also the subsequent impact on health outcomes for people with SCD in this region.

The framework shows four groups of factors that may influence healthcare service utilisation, namely: social determinants; predisposing factors; health system factors; and health status. It shows that predisposing factors such as age and educational level may act directly on the use of healthcare services or indirectly through their linkage to individuals’ social determinants like health beliefs and norms and influence their healthcare service use. Also, social determinants can influence the decision to use healthcare services directly or through their impact on health system factors and affect the decision and choice of healthcare service among people with SCD. Health status, like having a severe crisis or serious illness, affects the decision to use healthcare services including what kind of service to seek. The use of healthcare services and health status are interrelated. Thus, the use of healthcare services among people with SCD is influenced by different groups of factors through varied pathways, either directly or indirectly.

## Methods

### Study design

We employed a cross-sectional design to investigate the healthcare services usage by people with SCD. This afforded the study to collect quantitative data from a section of the people with SCD at a single point in time (October 1–29, 2021), providing a snapshot of healthcare service usage patterns and associated determinants over the last 12 months prior to the study [33].

### Study setting

The study was conducted in the Upper West Region of Ghana, one of the 16 administrative regions. Located in the North-Western corner of Ghana, it shared boundaries with Burkina Faso to the North, the Upper East Region to the East, the Northern Region to the South, and Cote D’Ivoire to the West. It covers a geographical area of 18,476 km^2^ (12.7% of the total land area of Ghana) [34]. The regional capital is Wa. There is a secondary-level health facility, the Wa Municipal Hospital, and 13 functional Community-based Health Planning and Services zones [34]. The study selected respondents from the Wa Municipal Hospital’s Sickle Cell Clinic which serves as a referral centre for people with SCD within the Region. The hospital is a 206-bed capacity facility with a staff strength of 11 medical doctors and 198 nurses while the SCD Clinic has 8 nurses including paediatric nurse specialists. The clinic provides out-patient and in-patient services with 580 SCD registrants and renders health education (SCD, medications, appropriate nutrition, and possible complications conditions), lab tests, and physical examination among others.

### Population

The study involved people living with SCD in the Upper West Region and accessing care at the Wa Municipal Hospital. Given the inclusion of children with SCD, caregivers were also recruited to provide responses on behalf of children unable to self-report. Participants were eligible if they had a confirmed diagnosis of SCD, were registered at the SCD clinic, resided in the region, and provided informed consent (or assent with parental consent for minors). However, those who were critically ill or hospitalised at the time of the study were excluded to prevent discomfort and ensure their abilities to effectively participate in the study. Additionally, individuals with SCD who are not registered for care at the hospital’s SCD clinic were excluded to maintain consistency in healthcare exposure across the sample.

### Sampling

The study sample size was determined using Yamane’s [35] formula: *n* =$\frac{N}{1+{N(\propto )}^{2}}$ Where n is the sample size, N is the population size (580 according to the hospital’s SCD 2021 register) and ∝ is the margin of error (0.05 at 95% confidence interval). Thus,

$$n=\frac{580}{1+{580(0.05)}^2}=236.7$$

Considering a 5% non-response rate, the sample size for this study totalled 248 people living with SCD.

Using the facility SCD register as the sampling frame, the study respondents were selected through a systematic random sampling technique. This register exclusively includes individuals who have been formally diagnosed with SCD at the hospital and enrolled in routine SCD care at its dedicated clinic. This technique ensured equal chances for each SCD registrant to be included in the study [36]. This was done by selecting registrants at random and subsequent registrants were selected using a regular interval of two (k = 580/248≈2). Thus, every second registrant after the randomly selected registrant was included until the sample size was obtained. Where a randomly selected registrant refused to participate, another registrant was randomly selected to fill in. While respondents were sampled from the SCD clinic register at Wa Municipal Hospital, healthcare service utilisation was assessed for SCD-related care at any healthcare facility, not exclusively the hospital. This distinction ensured that the study reflects broader care-seeking behaviours beyond a single facility while maintaining a consistent sampling frame.

### Procedures

A pre-tested questionnaire was used for the data collection (S1). It adapted from questions that addressed access, utilisation, and factors that influence healthcare use in previous studies [37, 38] and was based on a literature review. It was organised into four sections (A–D) to systematically capture key variables related to background information, healthcare accessibility and utilisation among individuals living with SCD through close-ended questions. Section A focused on socio-demographic characteristics, including variables such as sex, age (open-ended), education, marital status, occupation, and duration since diagnosis (open-ended). Section B collected information on health service accessibility, covering aspects like service availability, proximity, affordability, and provider availability. Section C assessed healthcare service utilisation, including frequency of emergency care, inpatient admissions, outpatient visits, and factors influencing care-seeking behaviours. Finally, Section D captured common health problems accounting for healthcare visits, documenting occurrences of painful crises, respiratory issues, infections, and other complications. The tool was pretested among 50 people living with SCD in the hospital’s SCD clinic to check for consistency and appropriateness in generating the needed data relevant to adequately addressing the research questions. It recorded a Cronbach Alpha reliability coefficient score of 0.71, indicating its internal consistency.

Through both telephone and face-to-face administration of questionnaires, the data were collected with the support of two research assistants. The research assistants were graduate students experienced in quantitative data collection and fluent in the local language (Waali). They were given a day’s training on the study’s purpose and tool before the data collection. Face-to-face administration of the questionnaire was done for those who were available at the facility. A telephone approach was conducted for respondents who consented to participate but were unavailable at the facility during the period of the data collection. For children under 15 years, caregivers or parents were recruited to provide responses on their behalf, ensuring accurate representation of the healthcare experiences of younger patients who were unable to self-report. Responses were recorded manually on paper-based forms. At the end of every data collection session, the questionnaires were checked for completeness and validity of responses.

### Study variables

The dependent variable of the study was healthcare service utilisation, measured by the use of any of the three healthcare services namely; emergency care, in-patient care, and non-preventive out-patient care. Preventive OP care, such as monthly SCD clinic visits, was excluded from the main healthcare service use measure to focus specifically on reactive care-seeking behaviours that reflect acute healthcare needs rather than routine disease management. Independent variables comprised predisposing factors such as sex, age, religion, marital status, educational level, and occupation; enabling factors mapped to healthcare accessibility consisting of availability of SCD-specific services, proximity to facilities, affordability of care, and availability of trained service providers; and duration of awareness of SCD status, reflecting individual knowledge and experience with managing the condition. Each of these factors was examined through specific questionnaire items to determine their influence on service utilisation.

### Data analysis

Data were entered into a template on Statistical Package for Social Sciences (SPSS) version 22.0 through a double-entry process to ensure good data quality. The dataset was then cleaned, re-coded, and prepared for analysis. Frequency, percentage, mean, and standard deviation were generated to summarise the data while inferential statistics (consisting of chi-square and logistic regression tests) were computed to examine associations between the dependent and independent variables. Before this, the age and duration of SCD diagnosis were transformed from discrete variables to categorical variables by converting them into age groups and year groups, respectively. Age was grouped into five groups, namely; < 10, 10–19, 20–29, 30–39, and 40 + years while duration/years with SCD diagnosis was categorised into six groups, namely; < 5, 5–9, 10–14, 15–19, 20–24, and 25 + years.

Health service accessibility was assessed using six items on a 4-point Likert scale (from 1–lowest to 4–highest accessibility). The six items evaluated were: perceived ease of accessing healthcare; availability of facilities providing SCD care; proximity of healthcare facilities to residence; travel time to reach healthcare facilities; affordability of SCD care services; and availability of capable health professionals for SCD care. It covered multiple dimensions, namely: the ability to identify healthcare needs; seek healthcare services; reach or obtain healthcare services; and fulfilment service needs [39]. All six items/variables were composited into a service accessibility index score by summing the responses of all the items. The total index score ranged between 6–24. The mean of the index scores was computed by dividing the sum total of the index scores (3,348) by the sample size (248) as 13.5 ± 2.127. Consequently, index scores < 13.5 were considered *“poor accessibility”* whereas those equal to or higher than 13.5 were graded *“good accessibility”* [40]. Healthcare service use was determined by a “yes” response to utilising at least one of the three healthcare services (emergency care, in-patient care, and non-preventive out-patient care) within the last 12 months. Non-preventive outpatient care refers to health facility visits made for symptom management, acute illness treatment, or follow-up for complications related to SCD, rather than routine check-ups or preventative screenings. Thus, healthcare utilisation was categorised as *“healthcare use”* indicating a use of at least one healthcare service and *“healthcare non-use”* indicating no use of any healthcare service.

Additionally, a chi-square test was carried out to ascertain the statistical association between health services utilisation and the independent variables (healthcare service accessibility and socio-demographic characteristics). Statistically significant variables were then considered for bivariate logistic regression (crude odds ratio). The variables that were statistically significant at this stage were then examined in the multivariate logistic regression (adjusted odds ratio). This test examines the relationship between the dependent variable and all the independent variables simultaneously, taking into account the effects on one another [41]. Statistical significance for all the inferential analyses was set at *p* < 0.05 at a 95% confidence interval.

### Ethical issues

The study obtained ethical clearance from the University of Cape Coast’s Institutional Review Board (Ref: UCC/IRB/A/2016/1108) and permission from the management of the Wa Municipal Hospital. Written informed consent was obtained from respondents aged 18 years or older while for those under 18 years, consent was obtained from a parent or guardian alongside the minor’s assent.. The respondents’ anonymity and confidentiality were ensured by using unique numbers rather than the personal identifying information of the respondents and securing the data on a password-protected personal computer of the principal investigator. Additionally, informed consent forms were separately stored in a locked filing cabinet to ensure confidentiality. The primary data files were backed up on a secure cloud storage platform to prevent data loss while maintaining restricted access. There was a stand-by clinical psychologist to provide psychological support if needed by any respondent during the data collection.

## Results

### Socio-demographic information of respondents

The socio-demographic characteristics of the 248 study respondents are presented in Table 1. Of these, 57.7% were female. The mean age of the respondent was 20.43 years with 31.9% aged less than 10 years and 11.9% aged 40 + years. More than half (51.6%) were Christians. Regarding marital status, 75.4% were single and 20.2% were married. Also, 14.5% had no formal education whereas 34.7% had primary, and 25.0% had tertiary education. Unemployed respondents were 61.7% while 16.1% were public/civil servants and 14.9% had other occupations. Also, 64.9% were rural residents. The mean years of diagnosis was 16.28 years with 27.8% having been diagnosed between 5–9 years ago, and 23.8% diagnosed 25 + years ago.

**Table 1:** Socio-demographic characteristics of study respondents

Socio-demographic variable	Frequency (*N* = 248)	Percentage (%)
Sex		
Male	105	42.3
Female	143	57.7
Age group (years)	Mean = 20.43 ± 15.928	
< 10	79	31.9
10–19	63	25.4
20–29	53	21.3
30–39	24	9.7
40 +	29	11.7
Religion		
Christian	128	51.6
Muslim	120	48.4
Marital status		
Single	187	75.4
Married	50	20.2
Widowed	11	4.4
Educational level		
No formal education	36	14.5
Primary	86	34.7
Junior High/Secondary	26	10.5
Senior High/Secondary	38	15.3
Tertiary	62	25.0
Occupation		
Unemployed	153	61.7
Farmer/breeder	10	4.0
Trader/business owner	8	3.2
Public/Civil servant	40	16.1
Other	36	14.9
Place of residence		
Rural	161	64.9
Urban	87	35.1
Duration of SCD diagnosis (years)	Mean = 16.28 ± 15.405	
< 5	53	21.4
5–9	69	27.8
10–14	23	9.3
15–19	25	10.1
20–24	19	7.7
25 +	59	23.8

### Healthcare service accessibility for people with SCD

Table 2 presents the SCD care accessibility experience of the respondents. It shows that over half (51.6%) found healthcare access difficult, while only 2.4% found it very easy. A vast majority (84.7%) reported limited availability of SCD care facilities. Affordability remained a major challenge, with 78.2% finding services expensive and encounters with SCD-trained professionals varied, with only 6% always meeting one.

**Table 2:** Accessibility to SCD care services

Accessibility Item	Frequency	Percentage
Easy or difficulty accessing a facility		
Very difficult	4	1.6
Difficult	128	51.6
Easy	110	44.4
Very easy	6	2.4
Availability of facility with SCD care		
Not available at all	2	0.8
Limited	210	84.7
Somewhat available	25	10.1
Available	11	4.4
Proximity to a health facility		
Very far	11	4.5
Far	170	68.5
Close	65	26.2
Very close	2	0.8
Time to facility		
Very long	45	18.2
Long	137	55.2
Short	64	25.8
Very short	2	0.8
Affordability of SCD service		
Very expensive	20	8.1
Expensive	194	78.2
Affordable	34	13.7
Frequency of meeting SCD-trained health professionals		
Seldom	13	5.2
Sometimes	118	47.6
Most times	102	41.2
Always	15	6.0

**Fig. 2:**
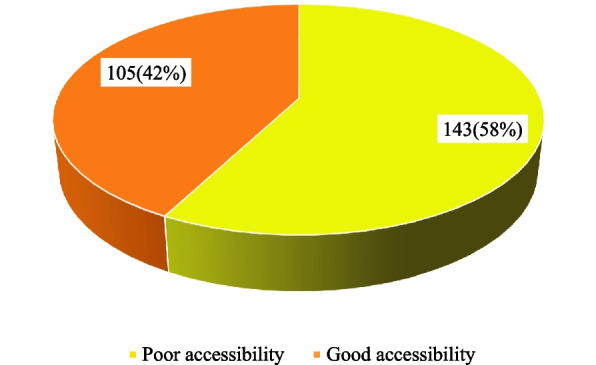
Overall accessibility to healthcare services providing sickle cell care among respondents

As shown in Fig. 2, 42% of the respondents had good accessibility to healthcare services providing sickle cell care in the region.

### Healthcare utilisation of people living with SCD

**Fig. 3:**
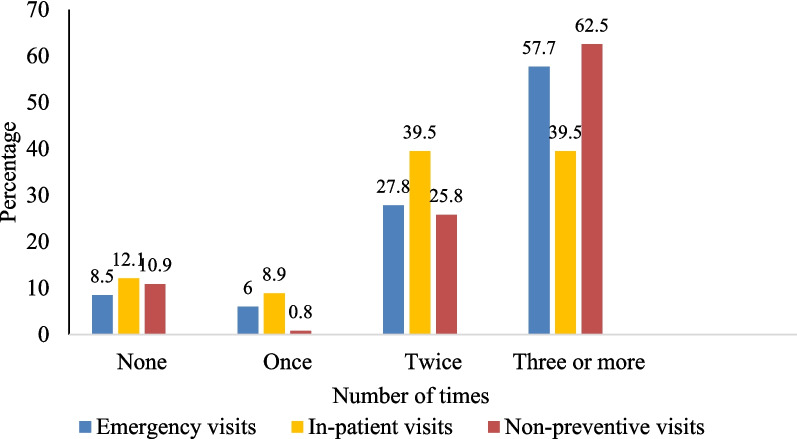
Health service utilisation among respondents

Healthcare services used within the last 12 months among the respondents are shown in Fig. 3. The services considered were categorised into emergency care, non-preventive outpatient department care, and in-patient care. More than half of the respondents used emergency care (57.7%) and non-preventive care (62.5%) three or more times in the last 12 months. Twice in the last 12 months, in-patient care (39.5%), emergency care (27.8%), and non-preventive out-patient care (25.8) services were used. Few respondents did not use emergency (8.5%), in-patient (12.1%), and non-preventive out-patient (10.9%) care in the year.

In all, as shown in Table 3, almost all the respondents (92.7%) used at least one healthcare service within the year. Common conditions leading to healthcare service use were painful crisis (81.7%), fever (78.3%), and mild/severe anaemia (46.5%). The significant sources of care for the most recent poor condition showed that while the majority (89.5%) accessed care at the hospital, few utilised self-medication (5.6%), prayers/healing services (0.8%) and traditional/herbal medication (0.8%).

**Table 3:** Diagnosis and health service utilisation among respondents

Health services use variable	Frequency (*N* = 248)	Percentage (%)
Overall health services use		
Use	230	92.7
Non-use	18	7.3
^a^Common conditions leading to health service use (***n*** = 230)		
Painful crisis	188	81.7
Fever	180	78.3
Anaemia	107	46.5
Pneumonia	74	32.2
Respiratory problem	51	22.2
Malaria	44	19.1
Cold and headache	25	10.9
Otitis media	21	9.1
Jaundice	4	1.7
Source of care in recent most poor health		
Hospital	222	89.5
Self-medication	14	5.6
Health centre	8	3.3
Prayers/Healing services	2	0.8
Traditional medicine	2	0.8

^a^Multiple responses

### Factors influencing healthcare utilisation of people with SCD

Reasons for the use or non-use of healthcare are shown in Table 4. Among those who used healthcare services, the reasons for the majority of them included having an NHIS subscription (94.3%) and proximity to a care facility (88.3%). Interestingly, 8.3% noted good quality of care as the main reason for seeking care. Among those who did not utilise any healthcare services, long waiting time (88.9%), NHIS non-subscription (72.2%), poor quality of care (72.2%), long distance to health facility (66.7%), and high cost of services (66.7%), among others, were reasons for most of them.

**Table 4:** Reasons for use/non-use of health service in recent illness

Reason	Frequency	Percentage (%)
^a^Reasons for use of health service (*n* = 230)		
NHIS subscription	217	94.3
Proximity to a care facility	203	88.3
Availability of logistics and drugs	172	74.8
Availability of health professionals	55	23.9
Good quality of care	19	8.3
Others	5	2.2
^a^*Reasons for non-use of health service (n* = 18)		
Long waiting time	16	88.9
Non-subscription of NHIS	13	72.2
Poor quality of care	13	72.2
Long distance to a health facility	12	66.7
High cost of service	12	66.7
Others	12	66.7
Lack of specialists	11	61.1
Lack of logistics	11	61.1

^a^Multiple answers

In Table 5, factors associated with healthcare service utilisation among the respondents are presented. The factors considered for the chi-square analysis included demographic characteristics and healthcare accessibility factors. The chi-square test showed that respondents’ age (*p* < 0.001), availability of health facilities (*p* = 0.025), closeness of health facility to residence (*p* = 0.005), and time taken to reach nearest health facility (*p* = 0.009) were significantly associated with healthcare service utilisation. In the multiple regression test, those aged 40 + years were 12.6 times (CI = 1.40–113.81, *p* = 0.024) more likely to utilise healthcare services than those aged less than 10 years. In addition, those who considered healthcare facilities to be very close to their residence were 97% less likely (AOR = 0.03, CI = 0.00–0.98, *p* = 0.026) to utilise healthcare services compared to those who considered it very far.

**Table 5:** Factors associated with healthcare service use among people living with SCD

Variable	Healthcare Service Usage		Chi-square (*p*-value)	COR(95%CI)*p*-value	AOR(95%CI) *p*-value
	**Use n(%)**	**Non-use n(%)**			
Sex			0.095(0.758)		
Male	98(93.3)	7(6.7)			
Female	132(92.3)	11(7.7)			
Age group (years)			21.08(<0.001)***		
< 10	76(96.2)	3(3.8)		Ref	Ref
10-19	61(96.8)	2(3.2)		1.11(0.11-11.07)0.932	1.49(0.13-17.25)0.750
20-29	48(90.6)	5(9.4)		0.92(0.08-10.55)0.945	1.14(0.08-15.60)0.921
30-39	17(70.8)	7(29.2)		2.92(0.32-26.25)0.340	2.92(0.31-27.78)0.351
40 +	28(96.6)	1(3.4)		11.53(1.30-102.02)0.028*	12.61(1.40-113.81)0.024*
Religion			3.30(0.069)		
Christian	115(89.8)	13(10.2)			
Muslim	115(95.8)	5(4.2)			
Marital status			4.76(0.092)		
Single	176(94.1)	11(5.9)			
Married	43(86.0)	7(14.0)			
Widowed	11(100.0)	0(0.0)			
Educational level			5.88(0.208)		
No formal education	34(94.4)	2(5.6)			
Primary	83(96.5)	3(3.5)			
JHS/JSS	25(96.2)	1(3.8)			
SHS/SSS	33(86.8)	5(13.2)			
Tertiary	55(88.7)	7(11.3)			
Main occupation			6.112(0.191)		
Unemployed	142(92.8)	11(7.2)			
Farmer/breeder	10(100.0)	0(0.0)			
Trader/business owner	6(75.0)	2(25.0)			
Public/Civil servant	36(90.0)	4(10.0)			

## Discussion

We assessed healthcare service utilisation among people living with sickle cell disease in the Upper West Region. It found that most of the respondents (58%) had poor accessibility to healthcare services providing sickle cell care. Also, it showed that more than half were regular users of emergency care, in-patient care, and non-preventive out-patient care in the last 12 months, resulting in almost all the respondents (92.7%) using at least one healthcare service within the period. In addition, it revealed that the predictors of healthcare use were being aged 40 + years while considering healthcare facilities to be very close to one’s place of residence decreased the odds of healthcare use. However, there were many barriers to healthcare service use, including long waiting times, long distances to health facilities, lack of means of transport, lack of specialists and care logistics, non-subscription of health insurance, poor quality of care, and high cost of service. Common negative health outcomes reported to account for hospital visits were pain crises, fever, acute respiratory problems, pneumonia and influenza, and acute otitis media.

Regarding the finding that most of the people with SCD experienced poor accessibility to healthcare services, this is consistent with the conclusions made in previous studies [42, 43]. The authors noted that poor access to appropriate health care is a major challenge for people living with SCD [43]. This is because people living with SCD mostly need comprehensive care and specialised care which are limited to tertiary-level health facilities [38]. Consequently, compared to others with chronic diseases like hypertension and diabetes, those with SCD experience more difficulty in receiving appropriate care [42]. They are thus unable to receive appropriate care to address crises and manage other complications associated with the condition.

Also, the finding that most people with SCD were regular users of emergency, in-patient and non-preventive care services is consistent with Hemker et al.’s finding that people with SCD are in constant need of healthcare services due to pain crises and other complications associated with the disease [44]. Similarly, this finding is in congruence with the established observation that people with SCD have a high use of healthcare services due to associated complications like vaso-occlusive pain crises [45]. It is noted that the likelihood of some common conditions and diseases resulting in life-threatening situations for this vulnerable population necessitates the need for prompt care, which could result in most people with SCD utilising more healthcare services than the general population [2, 14].

Due to poor health outcomes and associated complications of SCD, it is expected that most people with SCD will experience one health problem or another, necessitating the need for healthcare. Nevertheless, the study found some people with SCD do not utilise healthcare services, even when they experience some form of illness. It has been noted that people with SCD may not utilise healthcare services due to delays in being attended to and getting pain relief when they are sometimes told they are faking their pain or addicted to opioids [46]. Additionally, due to limited specialist facilities available to people with SCD, they are likely not to use healthcare services as noted by Miles et al. only to experience many barriers to care in non-specialist facilities [46]. Beyond financial constraints and accessibility challenges already identified in this study [47], delayed care-seeking exacerbates disease progression, leading to more complex health conditions that require specialized and costly interventions. This cycle of delayed treatment and worsening health outcomes further reinforces disparities in care access and affordability for individuals with SCD.

More so, the finding showed that those aged more than 40 years were more likely to use at least a healthcare service within the year. This is similar to the finding by Hamilton et al. where the population aged 40 and over had significantly more care visits yearly compared with those aged lower than 40 years [45]. Also, this is consistent with the finding in a study by Sanders et al., which concluded that the age of people with SCD was a significant factor in their healthcare utilisation [48]. Age has been reported to be a major determinant of healthcare use in people with SCD [16]. The current result may be due to increasing health issues as individuals age which may have directly or indirectly increased people with SCD’s vulnerability to experiencing SCD complications and other conditions such as diabetes and hypertension. Those aged 40 years and older may therefore have increased healthcare needs and use. Age is one of the socio-demographic factors explained in the conceptual framework by Andersen that influences care-seeking [30].

Additionally, the odds of utilising healthcare services were lower for those who considered their place of residence very close to a health facility. This is contrary to studies that have argued that being closer to a healthcare facility increases the chances that an individual will use healthcare services when sick in developing countries like Malawi, Ghana, Burkina Faso, and Kenya [49–52]. It may be explained as resulting from other barriers such as the health facility not having professionals trained in caring for people with SCD, poor quality of care, and high cost of service. This is in agreement with the argument that persons with SCD, particularly young adults, often visit the emergency department or health facilities for SCD-related concerns, probably because of an absence of primary care providers specialising in SCD [44].

Furthermore, the study identified some barriers to healthcare service utilisation among the respondents including long hospital waiting times, long distance to health facilities, lack of specialist care providers, poor care quality, and high cost of service despite the NHIS covering some SCD-related care such as hydroxyurea in a few selected facilities. Long distance to health facilities poses as a significant barrier to people living with chronic diseases including those with SCD as most of them experience a lack of transportation to the health facility in search of health care use [53].

Also, the finding that some people with SCD are faced with a lack of SCD-specialist care providers in close facilities, supports the finding by Renedo et al. that mostly only non-specialist care providers are available to provide care to people with SCD [54]. The finding is also in agreement with the observation made in previous studies that there is a lack of specialised SCD care providers in low- and middle-income countries and rural settings [55–57]. This comes against the backdrop that non-specialist care providers often do not have adequate understanding and knowledge of SCD and management and sometimes downplay risks [58].

Again, poor quality of care as a barrier to healthcare use among people with SCD has been reported in previous studies [14, 42, 54, 59]. In the current study, respondents cited poor quality as a reason for non-utilisation, which likely encompassed factors such as inadequate availability of specialized staff, long waiting times, insufficient medication or treatment options, and perceived inattentiveness of healthcare providers. This leads to a mistrust of care providers, amongst non-specialist staff especially, which may result from poor quality of care during emergency service and hospital admissions.

Another barrier observed in this study is the cost of healthcare services for people with SCD. This is consistent with findings establishing the high financial costs involved in obtaining care for most people with SCD [42, 60, 61]. This is because even those with health insurance subscriptions, their coverage is inadequate to cover the cost involved in seeking healthcare with health conditions linked to SCD status [14]. In Ghana, most of the complications associated with SCD are not covered under the National Health Insurance Scheme. Thus, most people with SCD experience catastrophic healthcare expenditures any time they seek healthcare. This is supported by the conceptual framework where Andersen notes that socio-economic factors which affect an individual’s ability to afford healthcare services influence their utilisation of care when unwell [30].

Most of the respondents experienced negative health outcomes such as painful crises, acute respiratory problems, pneumonia and influenza, and acute otitis media. The lives of persons with SCD are characterized by frequent and unpredictable pain episodes, and other complications of SCD like VOC, kidney, and spleen problems [62]. Several patient, provider, and health system factors contribute to the poor health outcomes of young adults with SCD during the transition [63–65].

### Implications of the study

Findings from this study highlight persistent gaps in healthcare accessibility for individuals living with SCD, reinforcing the need for policy-driven interventions to improve service delivery and coverage. While Wa Municipal Hospital remains the primary secondary-level facility for SCD diagnosis and management, respondents reported encountering barriers in accessing care across other facilities, including limited specialist availability, cost constraints, and inconsistent service provision. Policymakers should thus consider expanding specialist training programmes and deploying dedicated SCD management units beyond the Municipal Hospital to improve equitable access to specialist care [2, 66]. Again, expanding the integration of SCD-specific health coverage into the NHIS could reduce financial burdens associated with managing acute complications, ensuring that cost is not a deterrent to timely care-seeking [67].

Furthermore, at the healthcare facility level, findings emphasise the importance of improving provider knowledge and preparedness to manage SCD cases effectively. Training initiatives focused on early recognition of complications, acute pain management, and long-term disease monitoring could enhance care quality across various healthcare settings [2, 66]. Respondents frequently reported concerns about service accessibility, suggesting that targeted outreach programmes such as mobile clinics, telehealth consultations, and decentralised SCD management centres to bridge gaps in care availability for underserved populations [68]. Additionally, referral pathways between primary care centres and specialised facilities must be strengthened to facilitate smoother transitions for patients needing advanced interventions, ensuring continuity of care while reducing avoidable delays in treatment.

### Strengths and limitations

The study relied on self-reports which tend to overestimate or underestimate behaviours. As such, participants were encouraged to provide honest responses as much as possible. It may have suffered from recall bias as the respondents had to provide information on their health status and healthcare service use over 12 months. Also, the respondents included only those diagnosed and registered for SCD care at the Wa Municipal Hospital which is a referral centre within the region and thus, findings are not generalisable to people with SCD in the general population who do not access health care at the hospital. However, the findings provide some useful insight into what might happen to people with SCD in settings similar to the present study. The study’s use of a multi-dimensional accessibility index based on validated items provides a nuanced understanding of how accessibility factors impact service use.

## Conclusion

This study highlights significant barriers to healthcare accessibility among individuals with SCD, leading to delayed care-seeking and potential reliance on unapproved health measures. Such challenges increase the risk of late reporting of complications and preventable deaths. Also, the high healthcare utilisation observed within the year under study poor health status of this population, contributing to financial strain, reduced productivity, and deteriorating quality of life. Additionally, findings suggest that proximity to healthcare facilities alone does not ensure utilisation, as other accessibility challenges, such as long wait times, specialist shortages, and cost, play a critical role.

Additionally, the finding that considering one’s place of residence to be closer to a health facility is associated with a lower likelihood of healthcare utilisation suggests that other issues of accessibility may be more important in this population. Aside from only building health facilities closer to people, other issues hinder access to and use of health care such as long waiting times, inadequate specialists, and high cost of care among others. Should these barriers persist, Ghana may not achieve its sustainable development goal three target of reducing morbidities and deaths associated with chronic non-communicable diseases including SCD.

## Supplementary Information

Supplementary Material 1.

Supplementary Material 2.

## Data Availability

The dataset used and/or analysed during the current study is available as attachment.
