# Supplementary material for: Healthcare utilisation among people living with sickle cell disease in the Upper West Region of Ghana
Source: BMC Health Serv Res. 2025 Aug 25;25:1126. doi: 10.1186/s12913-025-13124-7 (PMC12376717; doi:10.1186/s12913-025-13124-7)
Supplement: Supplementary file 2 — Supplementary Material 2. [file 12913_2025_13124_MOESM2_ESM.pdf]

QUESTIONNAIRE: HEALTH SERVICES ACCESS, UTILISATION, HEALTH OUTCOMES AMONG PEOPLE LIVING WITH SICKLE CELL DISEASE

Questionnaire ID:_______________ **Date:_______/_____/2022**

**Section A: Socio-demographic Information**	**Response**
1. What is your sex?	1. Male<br>2. Female
2. How old are you	[………….] years
3. What religion do you practice?	1. Christian<br>2. Muslim<br>3. Traditionalist<br>4. Other………………………………
4. What is your marital status	1. Single<br>2. Married<br>3. Co-habiting<br>4. Divorce/separated<br>5. Widowed
5. What is your highest level of education?	1. None<br>2. Primary<br>3. JHS/JSS<br>4. SSS/SHS/Vocational<br>5. Tertiary
6. What is your main occupation?	1. Unemployed<br>2. Artisan<br>3. Farmer/breeder<br>4. Trader/businessman/woman<br>5. Public servant<br>6.Other………………………………
7. Where is your residence?	1. Rural<br>2. Urban
8. How long since you were diagnosed with Sickle Cell Disease?	[……….]

**Section B: Health service accessibility**	**Response**			
9. How easy or difficult do you feel about accessing health service	Very Difficult	Difficult	Easy	Very easy
10. How available are facilities providing sickle cell care to you?	Not available all	Limited	Somewhat available	Available
11. How close or far is a health facility providing sickle cell care to your residence?	Very far	Far	Close	Very close
12. How long does it take you to reach the health facility for care?	Very long	Long	Short	Very short
13. How affordable is the services provided for sickle cell care?	Very expensive	Expensive	Affordable	Very affordable

<table>
<tr><td>14. How often do capable health professionals available to care for you?</td><td>Seldom</td><td>Sometimes</td><td colspan="2">Most times</td><td colspan="2">Always</td></tr>
<tr><td colspan="7">Section C: Utilization of Health Services</td></tr>
<tr><td>15. Have you used any health facilities when sick?</td><td colspan="6">1. Yes
2. No</td></tr>
<tr><td>16. If yes, what kind of health facility did you use?</td><td colspan="6">1. Hospital
2. Clinic/Health centre
3. CHPS compound
4. Other: ……………</td></tr>
<tr><td>17. What factors influenced the use of this health facility? Tick all that apply</td><td colspan="6">1. Proximity
2. Availability of NHIS
3. Availability of Staff
4. Quality Service
5. Availability of logistics and drugs
6. Others, specify</td></tr>
<tr><td>18. If No, how do you treat yourself when you fall sick?</td><td colspan="6">1. Self-Medication
2. Prayers/Healing service
3. Traditional/herbal medicine
4. Others, Specify: ……………</td></tr>
<tr><td>19. What are your main reasons for not utilizing healthcare services when sick/had crisis? Tick all that apply</td><td colspan="6">1. Longer waiting time
2. Long distance
3. Poor quality service
4. High cost of service
5. Lack of NHIS
6. Lack of specialists
7. Lack of logistics and drugs
8. Health beliefs
9. Others, Specify</td></tr>
<tr><td colspan="7">How many times did you use the following services within the last 1 year for SCD related health issues?</td></tr>
<tr><td>20. a. Emergency care visits</td><td>0</td><td>1</td><td>2</td><td>3</td><td>4</td><td>5+</td></tr>
<tr><td>b. For what conditions was your visit?</td><td colspan="6">1. ………………………………
2. ………………………………
3. ………………………………</td></tr>
<tr><td>21. a. In-patient visits</td><td>0</td><td>1</td><td>2</td><td>3</td><td>4</td><td>5+</td></tr>
<tr><td>b. For what conditions was your visit?</td><td colspan="6">1. ………………………………
2. ………………………………
3. ………………………………</td></tr>
<tr><td>22. a. Non-preventive out-patient visits</td><td>0</td><td>1</td><td>2</td><td>3</td><td>4</td><td>5+</td></tr>
<tr><td>b. For what conditions was your visit?</td><td colspan="6">1. ………………………………
2. ………………………………
3. ………………………………</td></tr>
<tr><td>Section D: Health Outcomes</td><td colspan="6">Response</td></tr>
<tr><td>How many times did you have the following conditions within the last 1 year?</td><td>0</td><td>1</td><td>2</td><td>3</td><td>4</td><td>5+</td></tr>
</table>

23. Painful crises						
24. Acute respiratory problems						
25. Leg ulcers						
26. Renal complications						
27. Spleen problems						
28. Acute otitis media						
29. Pneumonia and influenza						
30. Fever						
31. Stroke						

END

Thank You.
